# Maternal obesity and its association with the mode of delivery and the neonatal outcome in induced labour: Implications for midwifery practice

**DOI:** 10.18332/ejm/85792

**Published:** 2018-04-12

**Authors:** Antonakou Angeliki, Papoutsis Dimitrios, Tzavara Chara

**Affiliations:** 1 Department of Midwifery, Midwifery School, ‘Alexander’ Technological Educational Institute of Thessaloniki, Greece; 2 Department of Obstetrics and Gynaecology, Shrewsbury and Telford Hospital NHS Trust, Shrewsbury, United Kingdom; 3 Department of Hygiene, Epidemiology and Medical Statistics, Medical School, University of Athens, Greece

**Keywords:** induction of labour, caesarean section, body mass index, primigravidae

## Abstract

**INTRODUCTION:**

Maternal obesity carries an increased risk of complications during pregnancy and childbirth. This study investigated whether the body mass index (BMI) of women with induced labour was associated with the mode of delivery and neonatal outcome.

**METHODS:**

This was a retrospective study of primigravidae women under the age of 40 years who were induced at term for various indications. Data were collected from the electronic database of the Maternity Unit where these women gave birth.

**RESULTS:**

We sampled 1274 women with a mean age of 26.3±5.9 years. The mean BMI at booking was 26.5 kg/m^2^, with 28.8% being overweight and 24.3% obese. In the sample, 70.4% had a normal vaginal delivery, 15.4% a caesarean section (CS) and 14.2% an instrumental delivery. An increasing BMI was independently associated with the odds for a CS, with women who were overweight and obese having a 1.58 and 2.75 times greater likelihood for a CS. The CS rate was 10.2% in women with a normal BMI, and increased to 15.8% for overweight and 24.9% for obese women (p<0.001). The increasing BMI did not affect the instrumental delivery rates in our cohort. The Apgar scores at one and five minutes were significantly lower in overweight and obese women compared to women with a normal BMI.

**CONCLUSIONS:**

We show that an increasing BMI in women with induced labour was associated with increased CS rates and lower Apgar scores. These findings highlight the important role of midwives in engaging women in weight management before they get pregnant.

## INTRODUCTION

According to the World Health Organization, women can be classified into four groups according to body mass index (BMI): underweight (<18.5 kg/m^2^), normal (18.5–24.9 kg/ m^2^), overweight (25.0–29.9 kg/m^2^) and obese (≥30.0 kg/ m^2^)^1^. There are reports that more and more women are overweight at the start of their pregnancy (BMI at booking ≥25 kg/m^2^) with rates ranging between 15% to 30%^2,3^. In England about half of women who are of childbearing age are either overweight or obese, with more than 15% being obese at the start of their pregnancy^4,5^.

Pregnant women who are obese at booking have an increased risk for complications, both for themselves and their babies during pregnancy and childbirth^6^. Women who are obese are at risk for gestational diabetes, miscarriage, preeclampsia, thromboembolism and postpartum hemorrhage^7,8^. There is a growing body of evidence that maternal obesity might represent an independent risk factor for an instrumental delivery^8,9^ and caesarean-section delivery^10^, and for adverse neonatal outcomes^11,12^ such as macrosomia, shoulder dystocia and stillbirth. The birthing choices of obese women may also be limited from restrictions in the use of birthing pools, on home births and the type of pain relief that can be given^6^. Moreover, after birth obese women are more likely to require additional support in breastfeeding due to difficulties in latching the baby on the breast^13^.

Women are more likely to have an induced labour due to associated complications of obesity during pregnancy^8^. In the United Kingdom, during 2011–2012, the rate of induction of labour in the general obstetric population was 22.1%, while during 2013-2014 it had risen to 25%^14^. Moreover, there are literature reports that the rates of induced labour are increasing worldwide^15^. It is thought that induced labour is less efficient than a spontaneous onset labour and therefore women who are induced are twice as likely to have a caesarean-section delivery^16^ or an assisted delivery^17^.

The aim of our study was to investigate the effect of the maternal BMI at booking with the mode of delivery and the neonatal outcome in women with induced labour. In order to account for the significant confounding factors of parity^3^, maternal age^18^, use of an epidural in labour^19^ and ethnicity^20^ on the success of induced labour, we restricted the inclusion criteria of our women to those who were primigravidae, under 40 years, of white-European ethnic background, and who did not use an epidural for analgesia during labour. We compare our findings against the evidence from existing literature, and highlight the significant role of midwives in the weight management of women before and during pregnancy.

## METHODS

This was a retrospective cohort study of women who were induced for various indications at term (gestational age ≥37 weeks) at the Maternity Unit, of the Shrewsbury and Telford Hospital NHS Trust, between January 2007 and December 2013.. The inclusion criteria that were applied included primigravidae-only women with singleton cephalic presentation deliveries, who were under 40 years old, without the use of epidural analgesia during labour and who self-reported that they were of white-European ethnic background. We selected these inclusion criteria because there is evidence that parity, increased maternal age, use of an epidural and non-white-European ethnic background are all risk factors that have a confounding effect on the outcome of induced labour^3,18-20^. Women who were induced for stillbirths, fetal congenital abnormalities and multiple pregnancies were excluded from the analysis. Data were collected from the obstetric electronic database of the Maternity Unit, and maternal features, labour and delivery characteristics, and neonatal data, were recorded.

The indications for the induction of labour were: post-dates pregnancy (gestational age at more than 41 weeks), reduced fetal movements, fetal growth restriction, hypertensive disorders of pregnancy (preeclampsia/eclampsia), diabetes mellitus (gestational or preexisting), and term pre-labour rupture of membranes for more than 24 hours. Other indications included intrahepatic cholestasis of pregnancy, maternal age over 40 years, and maternal request for social or mental health issues.

We included only women who had complete data for their body mass index (BMI) at booking. Other maternal features recorded were maternal age at delivery and their smoking status. Labour and delivery data included gestational age at birth, route of birth (normal vaginal delivery, instrumental vaginal delivery, caesarean section delivery) and liquor appearance (normal, meconium stained). The neonatal data that were recorded involved fetal gender (male, female), birth weight, head circumference, Apgar scores (at 1 and 5 minutes), cord gases taken at delivery (arterial/venous pH), and any possible admission to the neonatal unit.

Ethical approval for the collection and analysis of data in our study was obtained by the Research and Development Department of the Shrewsbury and Telford Hospital NHS Trust.

### Statistical analysis

Quantitative variables were expressed as mean values (and standard deviations) or as median values (and interquartile range), while qualitative variables were expressed as absolute and relative frequencies. For the comparison of proportions chi-squared tests were used, and the Mann-Whitney test was computed for the comparison of median values between two groups when the distribution was not normal. Univariate and multiple logistic regression analyses were used to find factors associated with the likelihood of having a caesarean section. Odds ratios (OR) with 95% confidence intervals (95% CI) were computed from the results of the logistic regression analysis. All reported p values were two-tailed. Statistical significance was set at p<0.05 and analyses were conducted using SPSS statistical software (version 19.0).

## RESULTS

Our sample consisted of 1274 women who had induced labour and a mean age of 26.3 years (SD=5.9). The mean BMI was 26.5 kg/m^2^ (SD=5.9), with 28.8% of the women being overweight and 24.3% obese. The percentage of women having a normal vaginal delivery was 70.4%, while 15.4% had a caesarean section and 14.2% an instrumental delivery (4.5% forceps, 8.9% ventouse, and 0.9% a dual instrumental delivery with forceps and ventouse). The indication for the CS delivery was recorded on the electronic database in 75% of the women in our sample, with failed induction of labour, failure to progress in labour, cardiotocographic abnormalities, and other indications such as maternal pyrexia, chorioamnionitis and placental abruption being 18%, 35%, 29.9% and 17.1%, respectively. There was meconium stained liquor in 15% of all deliveries and 4.5% of the newborns were admitted to the Neonatal Unit (Table 1).

**Table 1:** The demographic features of women and the labour, delivery and neonatal characteristics

	*Caesarean section*			
	*Total Sample N (%)*	*No N (%)*	*Yes N (%)*	*p*
Maternal age at delivery (years), mean (SD)	26.3 (5.9)	26.1 (5.9)	27.4 (5.8)	0.003^+^
Smoking				
No	1088 (86.8)	914 (84.0)	174 (16.0)	0.091^‡^
Yes	165 (13.2)	147 (89.1)	18 (10.9)	
BMI (kg/m2), mean (SD)	26.5 (5.9)	26.0 (5.7)	28.9 (6.9)	<0.001^+^
BMI				
Normal	598 (46.9)	537 (89.8)	61 (10.2)	<0.001^‡^
Overweight	367 (28.8)	309 (84.2)	58 (15.8)	
Obese	309 (24.3)	232 (75.1)	77 (24.9)	
Gestation in days, mean (SD)	278.6 (12.6)	279.4 (12.6)	276 (14.9)	0.001^+^
Route of birth				
Vaginal-normal	897 (70.4)			
Caesarean section	196 (15.4)			
Instrumental delivery	181 (14.2)			
Meconium stained liquor				
No	1077 (85.0)	931 (86.4)	146 (13.6)	<0.001^‡^
Yes	190 (15.0)	142 (74.7)	48 (25.3)	
Fetal Gender				
Female	621 (48.7)	533 (85.8)	88 (14.2)	0.242^‡^
Male	653 (51.3)	545 (83.5)	108 (16.5)	
Birth Weight (g), mean (SD)	3399.2 (576.2)	3368.5 (563.1)	3568 (618.6)	<0.001^+^
Head circumference at birth (cm), mean (SD)	34.8 (1.7)	34.7 (1.7)	35.3 (1.6)	<0.001^+^
Apgar score at 1 minute, median (IQR)	9 (8-9)	9 (9-9)	9 (8-9)	<0.001^++^
Apgar score at 5 minutes, median (IQR)	10 (10-10)	10 (10-10)	10 (9-10)	<0.001^++^
Cord gases taken at delivery Arterial pH, median (IQR)	7.24 (7.18-7.29)	7.22 (7.17-7.28)	7.25 (7.20-7.29)	0.001^++^
Cord gases taken at delivery Venus pH, median (IQR)	7.29 (7.24-7.33)	7.29 (7.25-7.33)	7.29 (7.23-7.33)	0.365^++^
Admitted to the Neonatal Unit (NNU)				
No	1005 (95.5)	853 (84.9)	152 (15.1)	<0.001^‡^
Yes	47 (4.5)	29 (61.7)	18 (38.3)	

+ Student t-test;

++ Mann-Whitney test;

‡ Pearson chi-squared test

**Figure 1:**
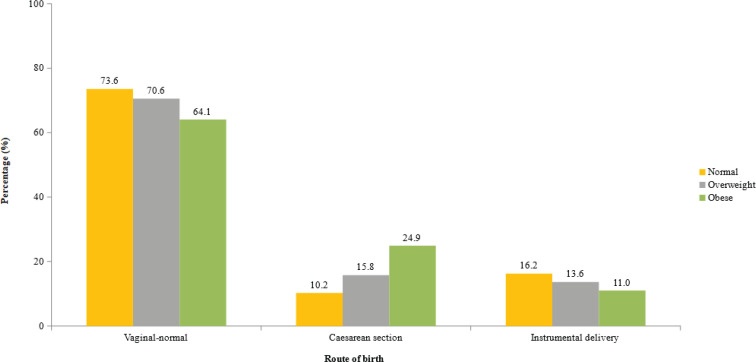
Proportion of women that had a normal vaginal delivery, caesarean section or instrumental delivery according to the BMI status

The proportion of women that had a normal vaginal delivery, caesarean section or instrumental delivery according to their BMI status is shown in Figure 1. There was a significantly increasing trend in the proportion of women who had a caesarean-section delivery with increasing BMI. The percentage of women who had caesarean section was 10.2% for a normal BMI, 15.8% for the overweight, and 24.9% for the obese (p<0.001). The percentage with instrumental delivery was 16.2% for a normal BMI, 13.6% for the overweight, and 11% for the obese, and was nonsignificantly different (p=0.481).

Table 2 presents the results from the univariate and multiple analyses with dependent variable the caesareansection rate. The univariate analysis showed that increased maternal age, gestational age, presence of meconium stained liquor, increased birth weight, increased head circumference and maternal BMI were associated with a greater risk for a caesarean section. Multiple logistic regression analysis showed that the presence of meconium-stained liquor, increased head circumference and increased maternal BMI were independently associated with the odds for a caesarean section. It was estimated that women with the presence of meconium-stained liquor had a 1.60 times greater likelihood for a caesarean section. Moreover, women who were overweight and obese had a 1.58 and 2.75 times greater likelihood for having a caesarean section, respectively.

**Table 2:** Results from the univariate and multiple analyses with dependent variable the caesarean section

	*Univariate analysis*		*Multiple analysis*	
	*OR (95% CI)+*	*p*	*OR (95% CI)+*	*p*
Maternal age at delivery (years), mean (SD)	1.04 (1.01-1.07)	0.003	1.03 (0.99-1.06)	0.058
Smoking				
No	1.00^++^		1.00^++^	
Yes	0.64 (0.38-1.08)	0.093	0.88 (0.51-1.53)	0.650
Gestation in days, mean (SD)	0.98 (0.9-0.99)	0.001	0.99 (0.98-1.01)	0.357
Meconium stained liquor				
No	1.00		1.00	
Yes	2.16 (1.49-3.12)	<0.001	1.60 (1.07-2.40)	0.023
Fetal Gender				
Female	1.00		1.00	
Male	1.20 (0.88-1.63)	0.242	1.12 (0.80-1.57)	0.506
Birth Weight (g), mean (SD)	1.07 (1.04-1.10)¶	<0.001	1.01 (0.97-1.06)¶	0.545
Head circumference at birth (cm), mean (SD)	1.27 (1.15-1.40)	<0.001	1.21 (1.05-1.39)	0.010
BMI (kg/m^2^)				
Normal	1.00		1.00	
Overweight	1.65 (1.12-2.43)	0.011	1.58 (1.06-2.34)	0.024
Obese	2.92 (2.02-4.23)	<0.001	2.75 (1.87-4.03)	<0.001

+ Odds Ratio (95% Confidence Interval);

++ indicates reference category;

¶ per 100 g

**Table 3:** Neonatal outcomes according to the BMI status of the mother.

*Total sample*	*BMI*				
	*Normal*		*Overweight/Obese*		*p*
	*Mean (SD)*	*Median (IQR)*	*Mean (SD)*	*Median (IQR)*	
Apgar score at 1 minute	8.5 (1.3)	9 ( -9)	8.4 (1.5)	9 (8-9)	<0.001^+^
Apgar score at 5 minutes	9.7 (0.8)	10 (10-10)	9.6 (0.8)	10 (9-10)	<0.001^+^
Cord gases taken at delivery Arterial pH	7.23 (0.08)	7.23 (7.18-7.29)	7.22 (0.09)	7.24 (7.18-7.28)	0.201^+^
Cord gases taken at delivery Venous pH	7.28 (0.07)	7.29 (7.24-7.33)	7.28 (0.07)	7.29 (7.24-7.33)	0.365^+^
Admitted to the Neonatal Unit, N (%)					
No	472 (96.1)		533 (95.0)		0.380^++^
Yes	19 (3.9)		28 (5.0)		
Normal deliveries					
Apgar score at 1 minute	8.7 (1.0)	9 (9-9)	8.6 (1.1)	9 ( -9)	0.069^+^
Apgar score at 5 minutes	9.7 (0.7)	10 (10-10)	9.8 (0.6)	10 (10-10)	0.374^+^
Cord gases taken at delivery Arterial pH	7.23 (0.08)	7.23 (7.16-7.30)	7.20 (0.10)	7.22 (7.15-7.28)	0.426^+^
Cord gases taken at delivery Venous pH	7.29 (0.05)	7.29 (7.25-7.32)	7.29 (0.07)	7.30 (7.25-7.33)	0.411^+^
Admitted to the Neonatal Unit, N (%)					
No	349 (97.5)		369 (97.9)		0.724^++^
Yes	9 (2.5)		8 (2.1)		

+ Mann-Whitney test;

++ Pearson chi-squared test

Table 3 shows the neonatal outcomes according to the BMI status of the mother. The pH values of the arterial and venous cord blood samples were not significantly different between women with a normal BMI and those who were overweight or obese. The proportion of neonates that were admitted to the Neonatal Unit was no different between women with a normal BMI and those who were overweight or obese. However, the Apgar scores at 1 and 5 minutes were significantly lower for babies born to women who were overweight or obese compared to those born to women who had a normal BMI (p<0.001). When the analysis was repeated for normal deliveries only, no significant differences were found.

## DISCUSSION

This study found that the increasing maternal BMI recorded at booking was independently associated with the odds for a caesarean section, with overweight and obese women presenting a 1.58 and 2.75 times greater risk for a caesareansection delivery, respectively. This finding is in line with other studies that showed that the increasing maternal BMI was also associated with an increased emergency CS delivery rate, which was 1.30 and 1.83 times greater for overweight and obese women compared to normal BMI women^11^. The pathophysiological reason that has been postulated to be behind the increased caesarean-section delivery rates is that the increased BMI, due to the adipose tissue being hormonally active, may predispose women to a reduced response to induced labour because of altered metabolic status when overweight or obese^21,22^. Moreover, it is not impossible that some of the alleged effect on the increased CS delivery rates could be attributed to the more frequent deconditioning in women with a high BMI^23^. A recent meta-analysis has shown that structured physical exercise during pregnancy can reduce the risk of a CS by almost 15%, probably through a significant reduction in overall weight gain in pregnancy^24^. Even though in our study we do not have any data on the physical activity of the women during pregnancy, we can hypothesize that being overweight and obese most probably reflects limited physical activity with subsequent increased weight gain in pregnancy and therefore a higher risk of CS.

In addition, other studies have reported that a high BMI is a risk factor for assisted delivery in both spontaneous and induced labour^9^. In contrast, our cohort study showed that the increasing BMI did not affect the instrumental delivery rates. We found differences in risk magnitude between caesareansection delivery in overweight/obese women and normal BMI women, and that maternal BMI did not influence the instrumental delivery rates. These findings can be explained by the different study designs reported in the literature, which involved women that were both nulliparous and multiparous for both spontaneous onset and induced labours^9,11^.

This study has also shown that increased head circumference at birth was an independent risk factor that was associated with an increased risk of a caesarean section (adjusted odds ratio=1.21). There are studies reporting that increased head circumference may lead to cephalopelvic disproportion and therefore to a caesarean-section delivery^25,26^. Moreover, the presence of meconium-stained liquor was also associated with a caesarean-section delivery in our cohort (adjusted odds ratio=1.60). There is evidence that the presence of meconium in the amniotic fluid is a function of the duration of labour and may rise from 2.8% in women prior to the onset of labour in an elective caesarean section to 23.1% in women in active labour^27^. It has been suggested that the presence of meconium is a sign and indicator of fetal hypoxia, associated with lower Apgar scores and higher rates of assisted delivery^27,28^.

### Implications for midwifery practice

In 2009 the Institute of Medicine (IOM) and the National Research Council (NRC) in the United States released a guideline on the recommended weight gain during pregnancy in relation to maternal pre-pregnancy BMI^29^. It was recommended that women with a normal BMI should gain no more than 35 lbs (or 16 kg) during pregnancy, overweight women should gain no more than 25 lbs (or 11.5 kg) and obese women no more than 20 lbs (or 9 kg). In the United Kingdom at present there are no formal, evidence-based guidelines from the UK Government or professional bodies on what constitutes appropriate weight gain during pregnancy^6^.

The IOM and NRC in September 2013 released an update reporting that many women still do not receive adequate preconception or post-conception advice about pregnancy-weight gain^30^. A very recent study in the United Kingdom demonstrated that midwives, who are considered the frontline professionals in the provision of weight-related advice to pregnant women, are still biased when providing advice to obese women by their own personal beliefs about body image and so their counselling is not always evidence-based^31^. Other studies have shown that UK professionals do not give information to women about the risks of obesity and the importance of weight management before or during pregnancy^13^.

According to the guidance from the National Institute for Health and Care Excellence issued in 2010, all health professionals involved in antenatal and postnatal services should engage women in dietary and physical activity interventions for weight management before pregnancy^6^. These interventions should include advice on dietary and behaviour change in order to maintain a healthy weight and also effective weight loss programmes by encouraging regular physical activity^6^. During pregnancy, dieting is not recommended and women should have around 200 calories more a day in the last trimester of pregnancy^6^. Also, moderateintensity physical activity will not harm the woman or her unborn child and is generally advised^6^.

There is currently no national guidance for UK professionals for weight management after childbirth. It has been suggested that managing a woman’s weight in the first few years after childbirth may reduce her risk of entering the next pregnancy overweight or obese^6^. Strategies that have been proposed involve having a healthy diet, taking a regular exercise and exclusive breastfeeding. The additional energy requirements of breastfeeding may help women return to their prepregnancy weight^32^. Moreover, if women are moderately active on a regular basis, this will not adversely affect their ability to breastfeed and could aid weight management^6^.

### Limitations and strengths

There are certain limitations to be considered in this study. First, this was a retrospective cohort study with the data being collected from an electronic database. This means that the accuracy of the final data was dependent on the practitioner entering each time the information on the database at the time of delivery. Second, we were unable to retrieve data about the induction of the labour process and the medications used, as this information is not recorded on the database and would therefore require manually retrieving the hospital notes for all women of the cohort, which logistically would be impossible. Third, the weight gain during pregnancy is not a mandatory field in the obstetric database that was used and therefore this information was missing.

The main strength of our study was that it included a large sample of women that generated statistically significant results comparable to those referenced in the literature for other countries. Moreover, the large sample size included women who were primigravidae, under 40 years old, white-European ethnic background, and without epidural use during labour, in order to account for the significant confounding factors of parity, maternal age, ethnicity and labour analgesia on the success of induced labour^3,18-20^.

## CONCLUSIONS

We have found that an increasing BMI in women with induced labour was associated with increased caesarean-section rates and lower Apgar scores. These findings highlight the important role of midwives as frontline health professionals in weight management before pregnancy.
